# *N*-Ethyl-*N*-phenyl-*p*-toluene­sulfonamide

**DOI:** 10.1107/S1600536810011219

**Published:** 2010-03-31

**Authors:** Islam Ullah Khan, Zeeshan Haider, Muhammad Nadeem Arshad, Sharafat Ali

**Affiliations:** a Materials Chemistry Laboratory, Department of Chemistry, GC University, Lahore, Pakistan

## Abstract

In the title compound, C_15_H_17_NO_2_S, the aromatic rings are oriented at a dihedral angle of 32.8 (1)°. The ethyl group and phenyl ring on the N atom adopt a staggered conformation with respect to the O atoms.

## Related literature

For related structures, see: Gowda *et al.* (2009 ▶); Nirmala *et al.* (2009*a* ▶,*b* ▶).
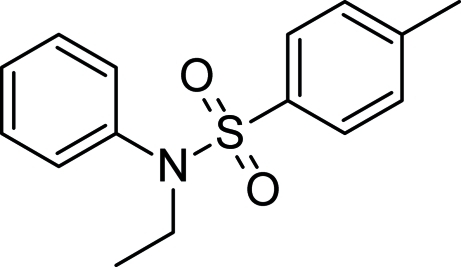


## Experimental

### 

#### Crystal data

C_15_H_17_NO_2_S
*M* *_r_* = 275.36
Orthorhombic, 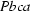

*a* = 14.1248 (6) Å
*b* = 10.4126 (5) Å
*c* = 19.7639 (10) Å
*V* = 2906.8 (2) Å^3^
*Z* = 8
Mo *K*α radiation
μ = 0.22 mm^−1^
*T* = 296 K
0.32 × 0.19 × 0.16 mm

#### Data collection

Bruker Kappa APEXII CCD diffractometer
Absorption correction: multi-scan (*SADABS*; Bruker, 2007 ▶) *T*_min_ = 0.951, *T*_max_ = 0.966
15016 measured reflections
3599 independent reflections
1759 reflections with *I* > 2σ(*I*)
*R*_int_ = 0.045

#### Refinement

*R*[*F* ^2^ > 2σ(*F* ^2^)] = 0.050
*wR*(*F* ^2^) = 0.157
*S* = 1.00
3597 reflections
173 parameters
H-atom parameters constrained
Δρ_max_ = 0.19 e Å^−3^
Δρ_min_ = −0.31 e Å^−3^

### 

Data collection: *APEX2* (Bruker, 2007 ▶); cell refinement: *SAINT* (Bruker, 2007 ▶); data reduction: *SAINT*; program(s) used to solve structure: *SHELXS97* (Sheldrick, 2008 ▶); program(s) used to refine structure: *SHELXL97* (Sheldrick, 2008 ▶); molecular graphics: *ORTEP-3 for Windows* (Farrugia, 1997 ▶) and *PLATON* (Spek, 2009 ▶); software used to prepare material for publication: *WinGX* (Farrugia, 1999 ▶) and *PLATON*.

## Supplementary Material

Crystal structure: contains datablocks I, global. DOI: 10.1107/S1600536810011219/ng2749sup1.cif

Structure factors: contains datablocks I. DOI: 10.1107/S1600536810011219/ng2749Isup2.hkl

Additional supplementary materials:  crystallographic information; 3D view; checkCIF report

## Figures and Tables

**Fig. 1.**
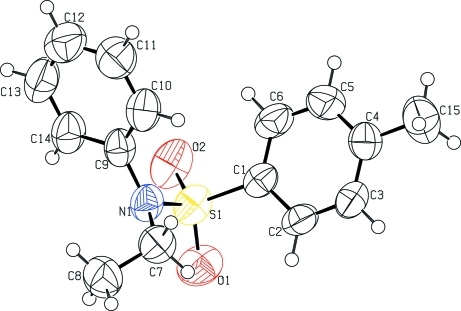
The labelled diagram of (I) with 50% probability level of drawn displacement ellipsoids.

**Fig. 2.**
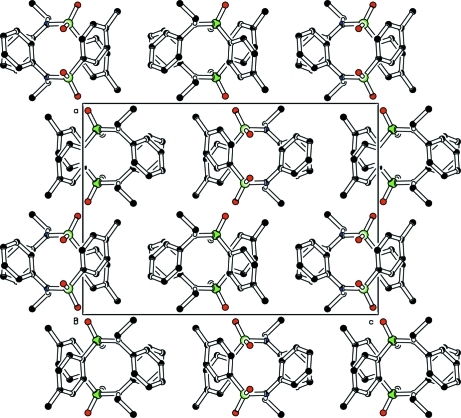
Unit cell packing for (I) Hydrogen atoms have been omitted for clarity.

## supplementary crystallographic information

### Comment

In recent literature the crystal structure of simple sulfonamide derivatives have been reported (Gowda *et al.*, 2009) II and (Nirmala *et al.*, 2009a,b) III & IV, which vary to the title compound (I) *N*-Ethyl-4-methyl-*N*-phenylbenzenesulfonamide in respect of ethylation at the nitrogen atom of I and substitution of methyl group at phenyl rings of III & IV. The dihedral angles between the both of the phenyl rings of all these four structures are not same as 32.79(0.10)° for I, 68.4 (1)° for II, 49.7 (1)° for III and 56.7 (3)° for IV, which may be due to substitution of alkyl groups at different position in all these molecules. The geometry around the sulphur atom S1 is distorted tertrahedral with the most distortion of 120.13(0.12)° for O1–S1–O2. No suitable hydrogen bonding have been found in the crystal structure of the molecule.

### Experimental

A mixture of 4-methyl-*N*-phenylbenzenesulfonamide (500 mg, 2.02 mmol), and sodium hydride (194 mg, 8.08 mmol) in *N*,*N*-dimethylformamide (10 ml) was stirred at room temperature for half an hour followed by addition of ethyl iodide (630 mg 4.04 mmol). Stirring was continued further for a period of three hours and the contents were poured over crushed ice. Precipitated product filtered, washed and recrystallized with methanol under slow evaporation for diffraction studies.

### Refinement

All the C—H H-atoms were positioned gemetrically and refined using a riding model with dC–H = 0.93 Å and *U*_iso_(H) = 1.2 *U*_eq_ for aromatic (C), with dC–H = 0.96 Å and *U*_iso_(H) = 1.5 *U*_eq_ for methyl (C), and with dC–H = 0.97 Å and *U*_iso_(H) = 1.2 *U*_eq_ for methylene (C). The two reflections 2 0 0 and 0 0 2 were omitted in final refinement

### Crystal data

**Table:**

C_15_H_17_NO_2_S	*F*(000) = 1168
*M**_r_* = 275.36	*D*_x_ = 1.258 Mg m^−^^3^
Orthorhombic, *P**b**c**a*	Mo *K*α radiation, λ = 0.71073 Å
Hall symbol: -P 2ac 2ab	Cell parameters from 2488 reflections
*a* = 14.1248 (6) Å	θ = 2.6–23.4°
*b* = 10.4126 (5) Å	µ = 0.22 mm^−^^1^
*c* = 19.7639 (10) Å	*T* = 296 K
*V* = 2906.8 (2) Å^3^	Needle, colorless
*Z* = 8	0.32 × 0.19 × 0.16 mm

### Data collection

**Table:**

Bruker Kappa APEXII CCD diffractometer	3599 independent reflections
Radiation source: fine-focus sealed tube	1759 reflections with *I* > 2σ(*I*)
graphite	*R*_int_ = 0.045
φ and ω scans	θ_max_ = 28.3°, θ_min_ = 2.1°
Absorption correction: multi-scan (*SADABS*; Bruker, 2007)	*h* = −18→10
*T*_min_ = 0.951, *T*_max_ = 0.966	*k* = −13→13
15016 measured reflections	*l* = −26→26

### Refinement

**Table:**

Refinement on *F*^2^	Primary atom site location: structure-invariant direct methods
Least-squares matrix: full	Secondary atom site location: difference Fourier map
*R*[*F*^2^ > 2σ(*F*^2^)] = 0.050	Hydrogen site location: inferred from neighbouring sites
*wR*(*F*^2^) = 0.157	H-atom parameters constrained
*S* = 1.00	*w* = 1/[σ^2^(*F*_o_^2^) + (0.0707*P*)^2^ + 0.2464*P*] where *P* = (*F*_o_^2^ + 2*F*_c_^2^)/3
3597 reflections	(Δ/σ)_max_ < 0.001
173 parameters	Δρ_max_ = 0.19 e Å^−^^3^
0 restraints	Δρ_min_ = −0.31 e Å^−^^3^

### Special details

**Table:**

Geometry. All esds (except the esd in the dihedral angle between two l.s. planes) are estimated using the full covariance matrix. The cell esds are taken into account individually in the estimation of esds in distances, angles and torsion angles; correlations between esds in cell parameters are only used when they are defined by crystal symmetry. An approximate (isotropic) treatment of cell esds is used for estimating esds involving l.s. planes.
Refinement. Refinement of *F*^2^ against ALL reflections. The weighted *R*-factor wR and goodness of fit *S* are based on *F*^2^, conventional *R*-factors *R* are based on *F*, with *F* set to zero for negative *F*^2^. The threshold expression of *F*^2^ > σ(*F*^2^) is used only for calculating *R*-factors(gt) etc. and is not relevant to the choice of reflections for refinement. *R*-factors based on *F*^2^ are statistically about twice as large as those based on *F*, and *R*- factors based on ALL data will be even larger.

### Fractional atomic coordinates and isotropic or equivalent isotropic displacement parameters (Å^2^)

**Table:**

	*x*	*y*	*z*	*U*_iso_*/*U*_eq_	
S1	0.12290 (5)	0.31259 (6)	0.45059 (3)	0.0736 (3)	
O1	0.03439 (13)	0.34190 (19)	0.48260 (10)	0.0982 (6)	
O2	0.14610 (15)	0.18289 (15)	0.43417 (9)	0.0940 (6)	
N1	0.12528 (13)	0.39272 (17)	0.37947 (10)	0.0670 (5)	
C1	0.21256 (17)	0.3761 (2)	0.50190 (10)	0.0605 (6)	
C2	0.19340 (18)	0.4774 (2)	0.54450 (11)	0.0678 (6)	
H2	0.1320	0.5089	0.5481	0.081*	
C3	0.2651 (2)	0.5321 (2)	0.58177 (12)	0.0759 (7)	
H3	0.2514	0.6004	0.6105	0.091*	
C4	0.35649 (19)	0.4880 (3)	0.57751 (12)	0.0739 (7)	
C5	0.3742 (2)	0.3868 (3)	0.53525 (15)	0.0852 (8)	
H5	0.4357	0.3554	0.5319	0.102*	
C6	0.3044 (2)	0.3305 (2)	0.49782 (13)	0.0773 (7)	
H6	0.3186	0.2617	0.4696	0.093*	
C7	0.09842 (18)	0.5301 (2)	0.38163 (13)	0.0760 (7)	
H7A	0.1544	0.5825	0.3749	0.091*	
H7B	0.0727	0.5502	0.4259	0.091*	
C8	0.0268 (2)	0.5621 (3)	0.32876 (15)	0.0934 (9)	
H8A	−0.0287	0.5106	0.3354	0.140*	
H8B	0.0528	0.5450	0.2848	0.140*	
H8C	0.0103	0.6513	0.3320	0.140*	
C9	0.19267 (18)	0.3535 (2)	0.32926 (11)	0.0625 (6)	
C10	0.2805 (2)	0.4097 (2)	0.32511 (13)	0.0773 (7)	
H10	0.2975	0.4741	0.3554	0.093*	
C11	0.3428 (2)	0.3706 (3)	0.27619 (15)	0.0926 (9)	
H11	0.4022	0.4086	0.2735	0.111*	
C12	0.3186 (3)	0.2762 (3)	0.23123 (15)	0.0975 (10)	
H12	0.3614	0.2502	0.1982	0.117*	
C13	0.2316 (3)	0.2209 (3)	0.23514 (14)	0.0941 (9)	
H13	0.2148	0.1569	0.2046	0.113*	
C14	0.1683 (2)	0.2588 (2)	0.28389 (13)	0.0782 (7)	
H14	0.1090	0.2204	0.2863	0.094*	
C15	0.4350 (2)	0.5510 (3)	0.61724 (16)	0.1138 (11)	
H15A	0.4907	0.4982	0.6154	0.171*	
H15B	0.4156	0.5613	0.6635	0.171*	
H15C	0.4487	0.6337	0.5981	0.171*	

### Atomic displacement parameters (Å^2^)

**Table:**

	*U*^11^	*U*^22^	*U*^33^	*U*^12^	*U*^13^	*U*^23^
S1	0.0876 (5)	0.0553 (4)	0.0779 (4)	−0.0172 (3)	0.0110 (4)	−0.0053 (3)
O1	0.0785 (13)	0.1021 (15)	0.1139 (15)	−0.0314 (10)	0.0304 (11)	−0.0179 (11)
O2	0.1434 (18)	0.0504 (10)	0.0881 (12)	−0.0189 (9)	0.0111 (11)	−0.0017 (8)
N1	0.0766 (13)	0.0560 (11)	0.0683 (12)	−0.0052 (9)	−0.0052 (10)	−0.0110 (9)
C1	0.0756 (17)	0.0524 (14)	0.0535 (12)	0.0029 (11)	0.0093 (11)	0.0042 (10)
C2	0.0684 (16)	0.0730 (16)	0.0621 (14)	0.0101 (12)	0.0143 (13)	−0.0062 (12)
C3	0.087 (2)	0.0846 (18)	0.0561 (13)	0.0084 (14)	0.0035 (13)	−0.0139 (12)
C4	0.0785 (19)	0.0872 (18)	0.0561 (13)	0.0058 (14)	−0.0046 (13)	0.0069 (13)
C5	0.080 (2)	0.094 (2)	0.0811 (18)	0.0276 (16)	−0.0046 (15)	0.0030 (15)
C6	0.097 (2)	0.0627 (16)	0.0723 (16)	0.0241 (14)	0.0094 (15)	−0.0060 (12)
C7	0.0835 (18)	0.0557 (15)	0.0887 (17)	0.0020 (11)	−0.0085 (15)	−0.0107 (12)
C8	0.100 (2)	0.093 (2)	0.0877 (19)	0.0131 (16)	−0.0141 (17)	0.0042 (15)
C9	0.0775 (17)	0.0494 (12)	0.0605 (13)	−0.0044 (11)	−0.0115 (12)	−0.0005 (10)
C10	0.094 (2)	0.0703 (16)	0.0677 (15)	−0.0120 (14)	−0.0053 (15)	0.0000 (12)
C11	0.090 (2)	0.107 (2)	0.0802 (19)	−0.0051 (17)	0.0048 (17)	0.0132 (18)
C12	0.117 (3)	0.107 (2)	0.0684 (19)	0.030 (2)	0.0103 (18)	0.0146 (17)
C13	0.132 (3)	0.082 (2)	0.0681 (18)	0.0105 (19)	−0.0103 (19)	−0.0157 (14)
C14	0.097 (2)	0.0623 (16)	0.0749 (17)	−0.0058 (13)	−0.0119 (15)	−0.0113 (13)
C15	0.099 (2)	0.148 (3)	0.094 (2)	−0.008 (2)	−0.0250 (19)	−0.006 (2)

### Geometric parameters (Å, °)

**Table:**

S1—O2	1.4271 (18)	C7—H7B	0.9700
S1—O1	1.4339 (19)	C8—H8A	0.9600
S1—N1	1.635 (2)	C8—H8B	0.9600
S1—C1	1.752 (2)	C8—H8C	0.9600
N1—C9	1.434 (3)	C9—C10	1.374 (3)
N1—C7	1.481 (3)	C9—C14	1.377 (3)
C1—C2	1.377 (3)	C10—C11	1.370 (4)
C1—C6	1.384 (3)	C10—H10	0.9300
C2—C3	1.375 (3)	C11—C12	1.368 (4)
C2—H2	0.9300	C11—H11	0.9300
C3—C4	1.373 (3)	C12—C13	1.360 (4)
C3—H3	0.9300	C12—H12	0.9300
C4—C5	1.368 (4)	C13—C14	1.372 (4)
C4—C15	1.509 (4)	C13—H13	0.9300
C5—C6	1.365 (4)	C14—H14	0.9300
C5—H5	0.9300	C15—H15A	0.9600
C6—H6	0.9300	C15—H15B	0.9600
C7—C8	1.492 (3)	C15—H15C	0.9600
C7—H7A	0.9700		
			
O2—S1—O1	120.13 (12)	H7A—C7—H7B	107.9
O2—S1—N1	106.42 (10)	C7—C8—H8A	109.5
O1—S1—N1	106.78 (12)	C7—C8—H8B	109.5
O2—S1—C1	108.83 (12)	H8A—C8—H8B	109.5
O1—S1—C1	107.13 (11)	C7—C8—H8C	109.5
N1—S1—C1	106.87 (10)	H8A—C8—H8C	109.5
C9—N1—C7	117.72 (19)	H8B—C8—H8C	109.5
C9—N1—S1	117.59 (15)	C10—C9—C14	119.5 (3)
C7—N1—S1	117.58 (16)	C10—C9—N1	121.3 (2)
C2—C1—C6	118.9 (2)	C14—C9—N1	119.3 (2)
C2—C1—S1	120.07 (19)	C11—C10—C9	119.7 (3)
C6—C1—S1	120.97 (19)	C11—C10—H10	120.2
C3—C2—C1	120.0 (2)	C9—C10—H10	120.2
C3—C2—H2	120.0	C12—C11—C10	120.8 (3)
C1—C2—H2	120.0	C12—C11—H11	119.6
C4—C3—C2	121.4 (2)	C10—C11—H11	119.6
C4—C3—H3	119.3	C13—C12—C11	119.5 (3)
C2—C3—H3	119.3	C13—C12—H12	120.2
C5—C4—C3	117.9 (3)	C11—C12—H12	120.2
C5—C4—C15	121.2 (3)	C12—C13—C14	120.4 (3)
C3—C4—C15	120.9 (3)	C12—C13—H13	119.8
C6—C5—C4	122.0 (3)	C14—C13—H13	119.8
C6—C5—H5	119.0	C13—C14—C9	120.1 (3)
C4—C5—H5	119.0	C13—C14—H14	120.0
C5—C6—C1	119.9 (2)	C9—C14—H14	120.0
C5—C6—H6	120.0	C4—C15—H15A	109.5
C1—C6—H6	120.0	C4—C15—H15B	109.5
N1—C7—C8	111.7 (2)	H15A—C15—H15B	109.5
N1—C7—H7A	109.3	C4—C15—H15C	109.5
C8—C7—H7A	109.3	H15A—C15—H15C	109.5
N1—C7—H7B	109.3	H15B—C15—H15C	109.5
C8—C7—H7B	109.3		
			
O2—S1—N1—C9	−33.36 (19)	C15—C4—C5—C6	−178.5 (3)
O1—S1—N1—C9	−162.81 (16)	C4—C5—C6—C1	0.2 (4)
C1—S1—N1—C9	82.81 (18)	C2—C1—C6—C5	−0.6 (4)
O2—S1—N1—C7	176.76 (17)	S1—C1—C6—C5	175.9 (2)
O1—S1—N1—C7	47.31 (19)	C9—N1—C7—C8	80.0 (3)
C1—S1—N1—C7	−67.08 (19)	S1—N1—C7—C8	−130.2 (2)
O2—S1—C1—C2	−155.91 (18)	C7—N1—C9—C10	56.3 (3)
O1—S1—C1—C2	−24.6 (2)	S1—N1—C9—C10	−93.6 (2)
N1—S1—C1—C2	89.5 (2)	C7—N1—C9—C14	−122.9 (2)
O2—S1—C1—C6	27.6 (2)	S1—N1—C9—C14	87.2 (2)
O1—S1—C1—C6	158.92 (19)	C14—C9—C10—C11	−0.3 (4)
N1—S1—C1—C6	−86.9 (2)	N1—C9—C10—C11	−179.5 (2)
C6—C1—C2—C3	0.4 (3)	C9—C10—C11—C12	0.2 (4)
S1—C1—C2—C3	−176.14 (18)	C10—C11—C12—C13	0.1 (5)
C1—C2—C3—C4	0.1 (4)	C11—C12—C13—C14	−0.2 (5)
C2—C3—C4—C5	−0.5 (4)	C12—C13—C14—C9	0.1 (4)
C2—C3—C4—C15	178.4 (2)	C10—C9—C14—C13	0.2 (4)
C3—C4—C5—C6	0.3 (4)	N1—C9—C14—C13	179.4 (2)
